# 1323. Efficacy and Safety of Telavancin Compared to Vancomycin for Cystic Fibrosis Pulmonary Exacerbation in Adults

**DOI:** 10.1093/ofid/ofab466.1515

**Published:** 2021-12-04

**Authors:** Mahmoud Shorman, ghassan wadi, Michael P Veve

**Affiliations:** 1 University of Tennessee, Knoxville, Tennessee; 2 University of Tennessee, Graduate Medical school, Knoxville, Tennessee; 3 Wayne State University, Detroit, Michigan

## Abstract

**Background:**

Telavancin (TLV) is an advanced generation lipoglycopeptide with activity against methicillin-resistant *Staphylococcus aureus* (MRSA), but there are limited patient outcomes in the setting of cystic fibrosis pulmonary exacerbation (CFPE). The study objective was to compare the efficacy and safety of TLV to vancomycin (VAN) in CFPE.

**Methods:**

Retrospective cohort conducted from 1/2011-6/2020. Inclusion criteria were: i) age ≥16 years, ii) hospitalized for CFPE with documented signs/symptoms of infection, iii) confirmed or suspected MRSA lower respiratory tract infection, iv) receipt of ≥48 hours of TLV or VAN. The primary outcome was 30-day CFPE-related readmission: infection recurrence, clinical worsening on treatment, or ADE requiring readmission. Secondary outcomes included adverse drug events (ADE) on therapy: acute kidney injury (AKI), rash, thrombocytopenias, cardiac abnormalities.

**Results:**

101 patients were included: 52 (52%) TLV, 49 (49%) VAN. The median (IQR) age was 22 (21-27) years, 50% were women, and 86% were Caucasian. The majority (84%) of patients had some federal health insurance; 19% had private health insurance. 93% of patients used a maintenance cystic fibrosis (CF) medication, and 35% had previous CF-therapy compliance concerns. 62% had a previous positive culture for MRSA; 22 (43%) TLV patients had documented MRSA infection on admission compared to 41 (84%) VAN (*P*< 0.001). The median (IQR) time to TLV initiation from admission was 1 (0.8-1.4) days. 13 patients were readmitted within 30-days due to CFPE; 8 (15)% TLV vs. 5 (10%) VAN (unAdjOR, 0.63; 95%CI, 0.19-2.1). Reasons for 30-day CFPE: TLV: 7 infection recurrence, 1 clinical worsening; VAN: 2 clinical worsening, 2 infection recurrence, 1 treatment-related ADE. When accounting for confounders, TLV was not associated with 30-day CFPE-related readmission (adjOR, 0.34, 95%CI, 0.1-1.2). Patients who received VAN more commonly experienced an ADE while hospitalized (18%), most notably AKI

**Conclusion:**

TLV was associated with similar short-term clinical outcomes compared to VAN for treatment of CFPE due to confirmed or suspected MRSA. Patients who received TLV experienced fewer overall ADEs.

**Disclosures:**

**Mahmoud Shorman, MD**, **Cumberland** (Grant/Research Support) **ghassan wadi, MD**, **Cumberland** (Grant/Research Support) **Michael P. Veve, Pharm.D.**, **Cumberland** (Grant/Research Support)**Paratek Pharmaceuticals** (Research Grant or Support)

